# Navicular Height by Weight-bearing Ultrasound: A Reliable and Valid Tool for Assessing the Medial Longitudinal Arch in Physically Active Adults

**DOI:** 10.1177/24730114261460463

**Published:** 2026-07-28

**Authors:** Natasha Noel-Barker, Charles Hillman, Ellys Pollon, Elizabeth Connors, Cameron Christie, William Pettitt, Jack Gallagher, Kathryn Higgins, Molly Riley, Thomas Bestwick-Stevenson, Stefan Kluzek

**Affiliations:** 1 School of Life Sciences, University of Nottingham, United Kingdom; 2 School of Medicine, University of Nottingham, United Kingdom; 3 Department of Orthopedic Surgery and Rehabilitation, Wake Forest School of Medicine, Winston-Salem, NC, USA

**Keywords:** medial longitudinal arch, navicular height, foot posture, ultrasonography, reliability

## Abstract

**Background:**

The medial longitudinal arch (MLA) height is an important clinical indicator of foot posture, linked to injury risk and rehabilitation targets. Assessment of navicular height (NH) in standing position is commonly used as a marker of MLA height. Methods such as palpation and imaging face limitations with operator variability, accessibility, and radiation exposure. Ultrasonography may be a practical alternative, but its reliability compared to established methods needs further evaluation. The research objective was to assess the reliability and validity of plantar ultrasonographic navicular height measures (US-NH) in standing position using a novel ultrasonography platform.

**Methods:**

A test-retest study design was adopted across 2 cohorts: cohort study 1 (CS1, 2023; n = 16) and cohort study 2 (CS2, 2024; n = 30), using the standing ultrasonography platform. Intra- and inter-rater intraclass correlation coefficients (ICCs) were assessed to evaluate method reliability. Validity was assessed using cadaveric dissection as a gold standard.

**Results:**

US-NH demonstrated excellent intra-rater reliability (ICC 0.956, 95% CI: 0.934-0.971), improving from good reliability in CS1 (ICC 0.827, 95% CI: 0.675-0.912) to excellent in CS2 (ICC 0.981, 95% CI: 0.969-0.989) after methodologic refinements. Inter-rater reliability also increased markedly, from poor in CS1 (ICC 0.459, 95% CI: 0.240-0.819) to excellent in CS2 (ICC 0.964, 95% CI: 0.940-0.978), indicating reduced operator-dependent variability. Dissection confirmed the bone visualised with weight-bearing ultrasonography corresponded accurately to the plantar navicular surface.

**Conclusion:**

Ultrasonography is a reliable, valid, and radiation-free method for measuring navicular height, in a physically active population. Dissections demonstrated the high validity of ultrasonographic assessment when compared to direct anatomical measurement.

**Level of Evidence:**

Level V, mechanism-based reasoning.

## Graphical abstract

**Visual Abstract:**
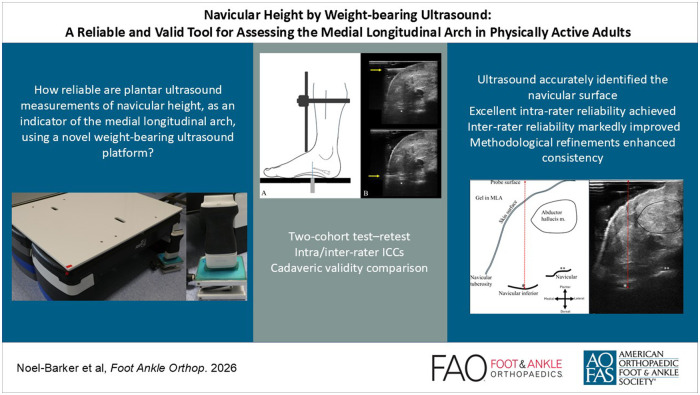

## Introduction

The medial longitudinal arch (MLA) is a key structure in load-sharing, as described by Kirby.^1^ A number of common musculoskeletal conditions have been associated with MLA height as a potentially modifiable foot posture marker^2^ including knee osteoarthritis,^3^ metatarsal stress fractures,^4^ and plantar fasciitis.^5^ Despite the foot’s 3-dimensional nature, clinical assessment often uses arch height as a simple and reliable indicator for assessment and as a rehabilitation target. The 6-Point Foot Posture Index (FPI-6), although deemed valid and reliable,^6^ provides only semiquantitative, subjective scores (−2 to +2), limiting detection of small changes during rehabilitation. Objective, continuous measures are needed for improved assessment and targeted rehabilitation outcomes.

Navicular height (NH) in standing position offers a unified MLA height measure, with positive correlations demonstrated between radiographic arch height. Although clinical NH measures such as normalised truncated NH demonstrate excellent reliability,^7^ these measures remain dependent on accurate manual landmark identification. Previous work has shown that the palpation of the navicular tuberosity lacks consistency^8^ and may vary with user experience,^9^ while also being influenced by variations in navicular morphology^10^ and weight-bearing conditions,^11^ thereby affecting reliability.^12^ Consequently, these factors may reduce sensitivity when detecting subtle changes over short time intervals or following assessment after interventions. As a result, discrepancies in NH measures persist, even with use of gold standard imaging.^13^

Standing ultrasonography offers a non-invasive, objective, and continuous measurement of NH in weight-bearing. Although this method has been assessed historically to observe the transverse arch,^14^ advances in probe technology have renewed interest. Shinohara et al^15,16^ demonstrated the navicular could be visualised with ultrasonography and although comparable to calipers and radiography, tester reliability and validity require further investigation.

This study assesses ultrasonography-based NH reliability via a test-retest study and validity through direct comparison with cadaveric dissection as a gold standard.

## Methods

### Population, Recruitment, and Sample Calculation

All participants provided written informed consent. The study recruited physically active bipedal participants aged 18-65 years. Physical activity was determined by self-reporting ≥150 minutes of moderate or ≥75 minutes of vigorous weekly exercise, based on the World Health Organization global recommendations.^17^ Exclusion criteria included recent lower limb injury or participation in another study within the past 3 months. Participants were screened by the primary investigator to ensure they met the inclusion criteria. Recruitment spanned two 30-day periods with 2 cohorts: cohort study 1 (CS1, 2023; n = 16) and cohort study 2 (CS2, 2024; n = 30). Sample size calculations for reliability^18^ required 51 feet (26 participants) to achieve an intraclass correlation coefficient (ICC) >0.75, with reliability estimated at 0.8 and precision 0.1 mm.

Participants attended 60-minute-long sessions involving physical examinations and repeated ultrasonography for reproducibility. Collected anthropometric and biomechanical data included age, foot length, truncated foot length (TFL) for standardisation,^11,19^ navicular height (NH) via palpation/ruler^20^ and plantar ultrasonography, joint range of motion, and foot posture index (FPI-6).^21^ A detailed table is provided in Table S1A.

### Equipment

**Figure 1.**
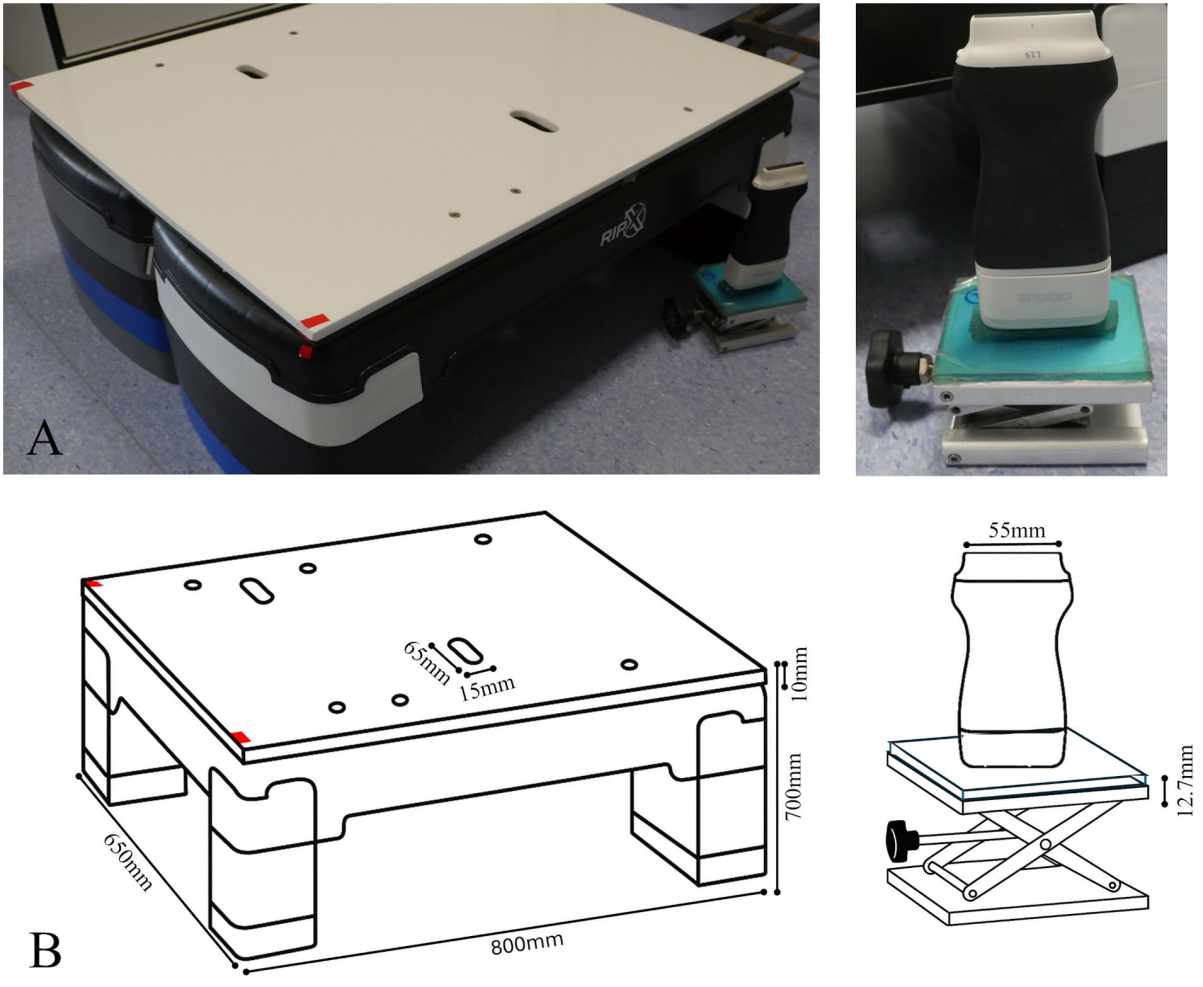
(A) Photograph of the custom platform and ultrasonographic device mounted onto a jack-lab stand to allow alignment of the transducer surface with the standing platform. (B) Schematic sketch of the custom plantar ultrasonographic platform with measurements. Further detail of individual components can be found in Table S2A.

A custom plantar weight-bearing ultrasonography device was built using 2 RIP-X step platforms with an opaque plastic cover and an integrated ultrasound port at the standing surface. A Clarius PAL HD Phased Linear Array probe (4-15 MHz, 50 mm depth, 165 mm width) was fixed to a Steel Lab Jack Stand Table (Fisher Scientific) using a 12.7-mm Orbital-Genie non-slip mat (Fisher Scientific), allowing the probe to sit flush with the platform surface (Figure 1). The probe was operated using the Plantar preset in the Clarius app (v9.0.0) on a Lenovo TB-J606F tablet. Images were downloaded in DICOM format and analysed using MicroDicom freeware (https://www.microdicom.com). Full standard operating procedures are provided in Supplement A.

### Ultrasonographic Navicular Height (US-NH) Measurement

**Figure 2.**
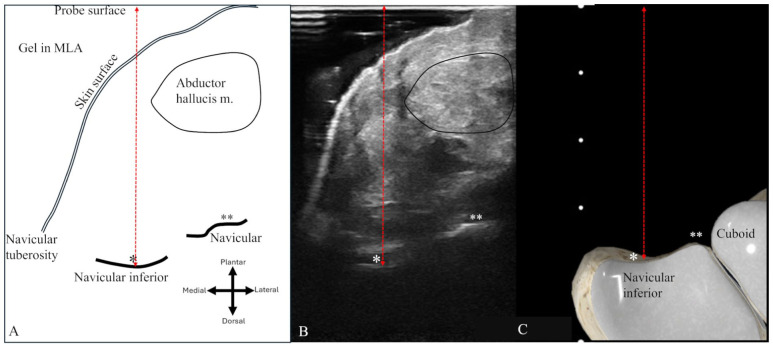
Schematic comparison of the medial foot’s cross-sectional anatomy in the coronal plane from the medial plantar side. (A) Schematic of key ultrasonographic anatomy; (B) ultrasonographic image using US-NH protocol; (C) 3D model of navicular-cuboid orientation. The single asterisk (*) marks the navicular’s most inferior surface; the double asterisk (**) marks the navicular inferior lateral surface; and the red dashed line indicates the vertical axis for US-NH measurement.

Plantar weight-bearing ultrasonographic navicular height (US-NH) produced an ultrasonographic image as displayed in Figure 2. The upper echogenic line represented the plantar skin surface with the medial border of the foot observed between the hypoechoic and anechoic regions. Measures were taken from the most inferior and flat surface of the navicular bone on the medial aspect of the MLA which can be identified by the hyperechogenic bone line. This is within localised concavity observed on imaging or anatomical inspection, typically lateral to the insertion site of the tibialis posterior tendon or adjacent to the plantar navicular tuberosity. Measures of US-NH were calculated as the maximum distance between the plantar skin and navicular surface. This distance was drawn using the vertical straight line distance tool in Microdicom and recorded in millimeters.

### Protocol

Participants stood barefoot in a relaxed stance, distributing weight evenly, with the ultrasound probe’s midline aligned to the longitudinal axis of the foot. Three navicular images were captured for each foot by 2 independent researchers, alternating between tester 1 and tester 2 (Figure S1A). Participants were required to step off the platform in between each measurement, thereby re-aligning between the participant and probe interface for each measure. Data files were stored independently for each measure to reduce bias between observers.

Two key protocol modifications were introduced in the second study phase (CS2) to improve ultrasonographic image acquisition (Table S3A). First, a circumferential seal of silicone molding filler (PP20-B) was applied around the transducer’s distal edge to stabilise gel (Skintact Ultrasonic Gel; Fanin Ltd) and reduce leakage. Secondly, a custom 3D-printed gel spacer (‘scoop’) localised and contained gel within the MLA via a shallow reservoir with low-profile walls, ensuring stable contact without altering foot alignment.

### Reliability Measures

Intra- and inter-rater reproducibility were assessed using ICCs by paired researchers, with 4 researchers in total. This design optimised reproducibility reflective of real-life application, encompassing both standardised ultrasonographic image acquisition and repeated measurement of NH each time. Researchers had minimal exposure to ultrasonography use beforehand and underwent training to ensure technique application in future environments.

Intra-rater ICCs were calculated between tester 1’s measures and inter-rater between tester 1 and tester 2 measures. Delayed intra-rater reliability was assessed for CS2, with 10 participants (20 feet) using the same captures re-measured at 4 weeks post initial data collection.

### Validity Measures: Cadaveric Model

To validate the US-NH measurements, 8 feet derived from 4 fresh frozen cadaveric specimens (4 right and 4 left, average foot length = 239 ± 10.77 mm) were assessed. Exclusion criteria included lower limbs with obvious deformities. The aim was to compare US-NH measures with the actual physical distance between the probe surface and the navicular bone, as marked with a hypodermic needle in direct contact with the bone. This measure is referred to as Direct NH.

**Figure 3.**
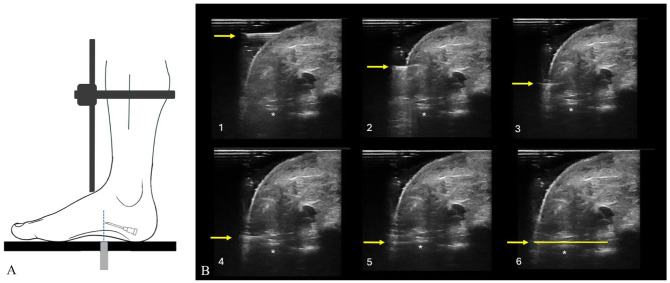
(A) Schematic representation illustrating the validation of US-NH measures using a cadaveric fresh frozen limb and ultrasonography-guided needle insertion. The foot is shown in a side profile with the ultrasound probe (grey) placed longitudinally beneath the medial arch. The needle is inserted from the medial aspect, out of plane with the ultrasound beam, allowing for accurate visualisation and targeted insertion at the high of the visualised navicular plantar border. (B) Six-piece ultrasonography series showing the plantar to dorsal movement of the needle (1 to 6; →, indicated with the yellow arrow) to the proposed navicular bone (*). The dashed yellow line indicates the final position of the inserted needle in contact with the navicular bone.

Limbs were sectioned at the knee and placed plantigrade on the custom ultrasonography platform. A custom rig and clamp stabilised each limb (Figure 3). The US-NH protocol was performed, locating the navicular bone, then inserting a 35-mm ultrasonography-guided hypodermic needle from the medial foot, and advancing superiorly until contacting the navicular surface (Figure 2). The perpendicular distance from the needle to the reference step was recorded as the Direct NH measurement, with adjustments for needle tip diameter. Dissection confirmed needle contact with the navicular bone, validating ultrasonography-guided placement. Deep dissection then revealed needle position relative to the navicular and identified structures including the abductor hallucis, tibialis posterior tendon, and long plantar and calcaneocuboid ligaments.

### Data Analysis

Given the sequential cohort design and protocol modifications between cohorts, analyses comparing or combining cohorts were conducted on an exploratory basis. Complete case analysis was performed to ensure that all statistical analysis was performed on complete paired data sets. Data normality was assessed both visually and using the Wilk test, with subsequent application of appropriate parametric or non-parametric tests. The primary reliability analysis for US-NH used the ICC coefficient with 95% CIs via a 2-way mixed effects model.^22^ ICC values were categorised according to Altman and Bland^23^: >0.90 (excellent), 0.75-0.90 (good), 0.5-0.75 (moderate), <0.5 (poor). Bland-Altman plots with limits of agreement (LOAs) defined as 1.5 ± 1.96 × SD^24^ were used to assess agreement. Measurement error and data reliability were estimated using the SE of the mean (SEM) and minimal detectable change (MDC). Further correlations were used to assess relationships between US-NH, palpated NH, and Direct NH. Statistical analyses were conducted using Python (version 3.10.6 for Windows; Python Software Foundation, 2025), in accordance with the Checklist for Statistical Assessment of Medical Papers guidelines.^25^ Cohort 1 and cohort 2 were analysed as separate groups and together to assess the difference in recruitment periods and methodologic improvements.

## Results

A total of 51 participants (102 feet) were screened and enrolled across both recruitment periods. However, because of lack of complete paired data, 5 participants from cohort 1 were excluded from the study results. Hence, 46 participants, corresponding to 92 feet, and recruitment periods (CS1: 32 feet; CS2: 60 feet), are presented in the results. The distribution of participants assigned female and male at birth was equal. The overall median age was 21 years (IQR: 20-21), with females being, on average, 2 years older than males. Detailed demographic and clinical characteristics for the overall study population and each recruitment cohort are presented in Table 1. Participants in CS2 were older, with a median age of 25.5 years (IQR: 21-50) compared with 20 years (IQR: 20-21) in CS1. CS1 participants presented as shorter (166.50 ± 7.95 cm) and with a higher body mass index (BMI) (25.11 [IQR: 23.69-27.64]) compared to CS2 (height: 174.20 ± 8.80 cm, BMI 23.01 [IQR: 21.54-25.03]).

**Table 1.** Participant Demographics and Navicular Height Data.^a^

	Total Cohort			CS1			CS2		
	All	Male	Female	All	Male	Female	All	Male	Female
Number of feet (%)	92 (100)	46 (50)	46 (50)	32 (34.8)	14 (43.8)	18 (56.2)	60 (65.2)	32 (53.3)	28 (46.7)
Age, y	21.00 (20-22)	20.00 (20-21)*	21.00 (20-30.5)*	25.50 (21-50)*	21.00 (19-24)	34.00 (26-53)*	20.00 (20-21)*	20.00 (20-21)	21.00 (20-21)*
Height, cm	171.52 (9.24)	176.88 (8.48)*	166.17 (6.49)*	166.50 (7.95)*	171.86 (8.08)*	162.33 (4.83)*	174.20 (8.80)*	179.08 (7.78)*	168.63 (6.27)*
Weight, kg	71.10 (63.50-78.55)	75.50 (70.05-82.79)*	64.00 (61.38-74.30)*	72.25 (63.88-79.25)	78.00 (72.12-84.22)	64.00 (62-75)	70.75 (63.35-78.55)	74.80 (68.8-81.06)	64.30 (61.2-72.2)
BMI	23.61 (22.15-25.95)	23.81 (22.12-25.38)	23.47 (22.24-25.96)	25.11 (23.69-27.64)*	25.38 (24.01-30.77)*	24.84 (23.32-25.96)*	23.01* (21.54-25.03)*	23.30 (21.51-25.07)*	22.54 (21.68-24.36)*
TFL, mm	186 ± 12.4	193 ± 12.2*	179 ± 7.76*	183 ± 9.77*	188 ± 9.04	178 ± 7.99	188 ± 13.2*	195 ± 13	180 ± 7.68
US-NH, mm	30.8 ± 7.57*	30.6 ± 6.76	30.9 ± 8.37	27.6 ± 5.5*	27.5 ± 5.59*	27.7 ± 5.6*	32.4 ± 8.01*	32 ± 6.86*	33 ± 9.26*
US-NH/TFL	0.166 ± 0.043	0.16 ± 0.0395	0.172 ± 0.0463	0.151 ± 0.0311*	0.146 ± 0.0303	0.155 ± 0.032*	0.174 ± 0.0469*	0.165 ± 0.042	0.183 ± 0.0511*
NH, mm	33.4 ± 8.49*	33.5 ± 7.63	33.4 ± 9.35	34.6 ± 9.51	37 ± 8.37*	32.8 ± 10.2	32.8 ± 7.9	32 ± 6.87*	33.8 ± 8.97
NH/TFL	0.202 ± 0.212	0.175 ± 0.0454	0.228 ± 0.295	0.25 ± 0.352	0.197 ± 0.0474*	0.292 ± 0.469	0.176 ± 0.0464	0.165 ± 0.0417*	0.188 ± 0.0493

Abbreviations: BMI, body mass index; NH, navicular height (palpated); TFL, truncated foot length; US-NH, navicular height (ultrasonography).

a Medians (IQRs) are shown for non-parametric variables such as age, weight, and BMI; means (SDs) are reported for parametric data. Navicular height is reported using ultrasonography (US-NH) and palpated methods (NH), along with ratios to truncated foot length (TFL). Data are presented for the full sample, by cohort, and by assigned sex at birth.

* Statistical significance (*P* < .01).

### Ultrasonography vs Palpated Navicular Height

Average US-NH across the study was 30.8 ± 7.57 mm with CS1 presenting significantly lower values (27.6 ± 5.5 mm) than CS2 (32.4 ± 8.01 mm) (*P* < .01). Palpated NH measures averaged 33.4 ± 8.49 mm across the entire study population, with a significantly higher NH observed by CS1 males (≈5 mm higher) than CS2 males (*P* < .01). US-NH measures were on average −2.67 mm (*r* = 0.71, CI: 1.39-3.95) lower than palpated NH, with a greater difference observed in CS1, −6.79 mm (*r* = 0.41, CI: 3.68-9.91), than CS2, −0.35 mm (*r* = 0.98, CI: −0.04 to 0.76). Small consistent changes were noted in the ratios of US-NH/TFL and NH/TFL, with CS1 exhibiting a lower ratio by 0.074.

### Reliability of Ultrasonographic Measurements

#### Intra-rater reliability

The intra-rater reliability of US-NH across the total study population was classified as ‘excellent’, with an ICC of 0.956 (95% CI: 0.934-0.971; Table 2). Variation was observed between cohorts where CS1 had ‘good’ reliability (ICC 0.827, 95% CI: 0.675-0.912), improving to ‘excellent’ in CS2 (ICC 0.981, 95% CI: 0.912-0.989). Delayed intrarater reliability, assessed only in CS2, remained ‘excellent’ (ICC 0.979, 95% CI: 0.948-0.992) despite a slight decrease in the lower bound.

**Table 2.** ICC Single Measures Analysis for Both Study Iterations.^a^

Study Cohort	Total Cohort		CS1		CS2		
	Intra-rater	Inter-rater	Intra-rater	Inter-rater	Intra-rater	Inter-rater	Delayed intra-rater
ICC	0.956*	0.864*	0.827*	0.459*	0.981*	0.964*	0.979*
95% CIs	0.934, 0.971	0.802, 0.908	0.675, 0.912	0.240, 0.819	0.969, 0.989	0.940, 0.978	0.948, 0.992
SEM, mm	0.466	1.438	0.108	4.374	0.215	0.403	0.322
MDC, mm	1.291	3.986	0.300	12.123	0.595	1.117	0.892

Abbreviations: ICC, intraclass correlation; MDC, minimal detectable change; SEM, SE of the mean.

a ICC results are presented for intra-rater reliability (observer T1) and inter-observer reliability (between observer T1 and T2). Results include lower and upper 95% CIs (CI). Delayed intra-rater reliability was assessed using a sampled re-measure 4 weeks after the initial capture (n = 20 feet).

* Statistical significance (*P* < .01).

The SEM and MDC across the total cohort was 0.466 mm (1.7%) and 1.291 mm (4.8%) of the mean US-NH. Cohort differences were observed again with the SEM increasing from 0.108 mm (0.4%) in CS1 to 0.215 mm (0.8%) in CS2. Similarly, the MDC significantly increased from 0.3 mm (1.2%) in CS1 to 0.595 mm (2.1%) in CS2 (*P* < .01). Delayed intra-rater reliability showed further significant increases to SEM 0.322 mm (1.1%) and MDC 0.892 mm (3.2%) based on the mean US-NH CS2 value (*P* < .01).

**Figure S3B.**
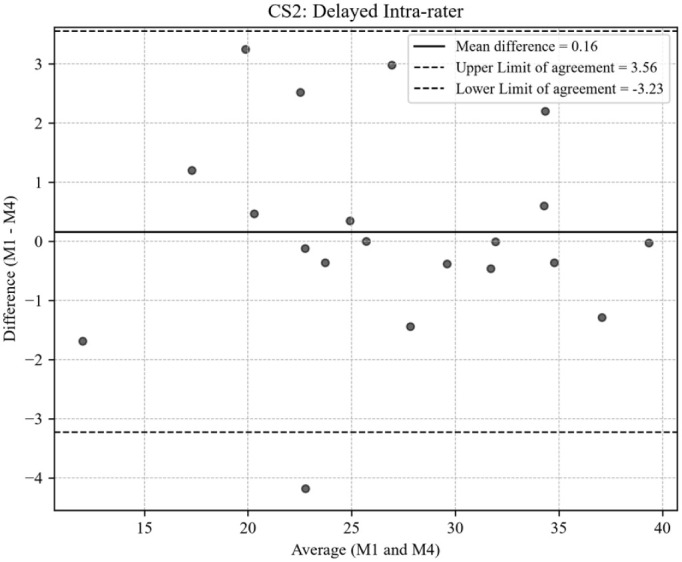
Bland-Altman plot showing intra-rater reliability of US-NH measures (mm) as recorded by Tester 1 (measures 1 and 4)in cohort 2 (CS2) Delayed.

**Figure S4B.**
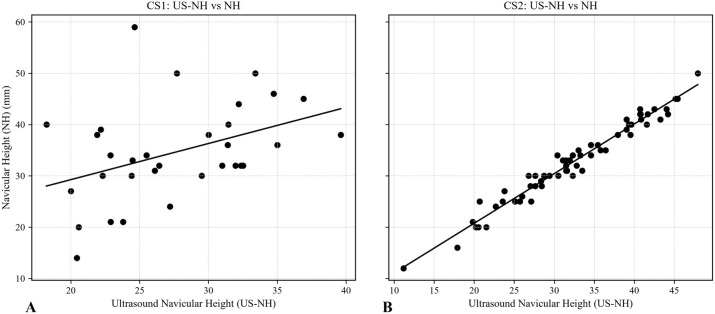
Bland-Altman plot showing inter -rater reliability of US-NH measures (mm) as recorded between Tester 1 and Tester 2 (measures 1 and 2) in Cohort 1 (CS1).

**Figure S5B.**
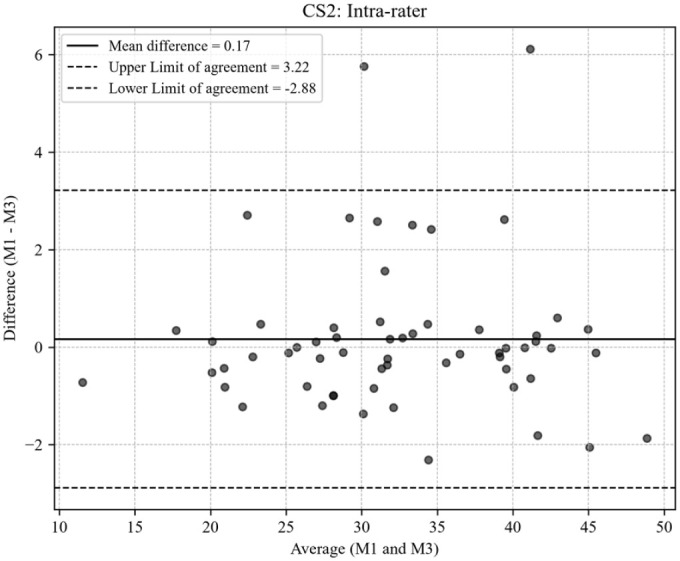
Bland-Altman plots showing inter-rater reliability of US-NH measures (mm) as recorded by Tester 1 and Tester 2 (measures 1 and 2) in Cohort 2 (CS2).

Bland-Altman plots demonstrated that a small variation was observed between intra-rater measures, observed as −0.42 ± 3.1 mm. CS2 had smaller variation, averaging 0.17 mm ± 1.56 mm (−2.86 mm −3.19 mm). Two measures fell outside the LOA, with US-NH values between 30 and 42 mm. Delayed tester 1 measures averaged 0.16 mm higher than the first collected value, with only 1 data point falling outside the LOA with an US-NH of 22.5 mm. Bland-Altman plots are provided in the supplement (Figures S1B-S5B).

#### Inter-rater reliability

Inter-rater reliability for the total cohort was ‘good’, with an ICC of 0.864 (95% CI: 0.802-0.908). Similarly, cohort variation was noted, with CS1 presenting ‘poor’ reliability (ICC 0.459, 95% CI: 0.240-0.819) and then improving to ‘excellent’ in CS2 (ICC 0.964, 95% CI: 0.940-0.978). The overall total cohort SEM and MDC are greater than intra-rater with 1.439 mm and 3.986 mm representing approximately 5.4% and 14.9% of mean US-NH. Large cohort differences exist: within CS1, the SEM was indicated as 4.374 mm (17.6%) and the MDC was 12.123 mm (48.9%), whereas CS2 values significantly reduced to an SEM of 0.403 mm (1.4%) and an MDC of 1.117 mm (4%) (*P* < .01).

Bland-Altman plots demonstrated a mean difference of –0.64 ± 5.66 mm with LOAs between –12.11 and 10.83 mm. Significant improvement was noted between CS1 and CS2 with narrower LOAs (–3.74 to 4.51 mm) and mean difference 0.38 ± 2.12 mm (*P* < .01). CS1 had 2 data points below the lower LOA (US-NH 26-33 mm), whereas CS2 had 3 above the upper LOA and 1 below (31-36 mm). CS2 values clustered below the mean bias, especially between 16 and 46 mm US-NH.

### Validity: Direct Cadaveric Measures

**Figure 4.**
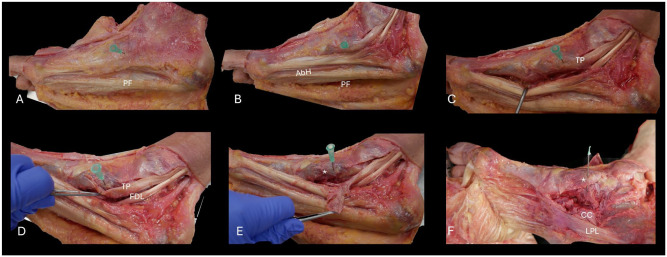
A sequence of cadaveric images of same specimen, showing the location of the hypodermic needle in relation to other anatomical structures: abductor hallucis longus muscle (ABh), flexor digitorum longus (FDL), long plantar ligament (LPL), navicular bone (*), plantar calcaneocuboid ligament (CC), plantar fascia (PF), tibialis posterior (TP). Sequential dissection started with (A) skin reflection to show PF, (B) removal of fascia over ABh, (C) detachment of ABh from the first metatarsal, (D) detachment of medial ankle ligaments, (E) detachment of TP from navicular and plantar attachment sites, and (F) the needle is shown touching the second prominence of the navicular bone.

A needle was inserted to contact the medial plantar surface of the navicular bone in 14 cadaveric models (7 donors). Direct measurement of needle height above the probe surface served as the gold standard and was compared with ultrasonographic measurements. Figure 4 demonstrates the needle insertion, ultrasonographic assessment, and direct measurement after dissection. Dissection confirmed needle contact with the navicular bone base (Figure 4). Paired comparisons of NH, US-NH, and direct cadaveric measurements (Direct NH) showed strong correlations (*r* = 0.98-0.99, *P* < .05). With the numbers available, no significant difference could be detected between US-NH and Direct NH (mean difference 0.95 mm; 95% CI: –4.04 to 5.94 mm), indicating good agreement; however, US-NH values were on average 3.88 mm lower than NH (95% CI: –5.51 to –2.26 mm).

## Discussion

This study demonstrated excellent reliability and validity of plantar ultrasonography-derived navicular height (US-NH). Moreover, it indicated that the technique possesses precision and sensitivity potentially useful in rehabilitation and screening contexts. It offers an objective means of assessing the MLA, including potential for monitoring temporal changes in arch height following injury or throughout the rehabilitation process. By incorporating methodologic refinements across 2 study phases, the work discusses the reliability and validity of novel measures on and the impact of protocol changes on reliability measures. To enhance reproducibility and practical utility of this technique, this article details construction, standard operating procedures, and protocol refinements for the ultrasonography platform (Table S3A).

### Reliability

US-NH showed strong consistency when measured by the same person, improving from good in CS1 (ICC = 0.827) to excellent in CS2 (ICC = 0.981). Inter-rater reliability also improved from poor to excellent after introducing a custom gel-spacer and better transducer support that reduced user-related variation. Ultrasonography can reliably identify the navicular because of strong acoustic contrast between bone and soft tissue, allowing clear visualisation of the navicular edge.^26,27^ Despite minimal experience, all researchers successfully identified the navicular, after training, supporting the methods usability. The ICCs observed align with existing NH literature reporting coefficients ranging from 0.78 to 0.98 across a variety of methods including MRI and weight-bearing CT,^28-31^ noting that weight-bearing^11,30^ and/or tester experience^11,32^ can influence reliability. When directly comparing to low-cost and non-invasive NH tests, the narrow CIs observed in the present study, particularly in CS2, further indicate reduced uncertainty and improved stability compared with the wider variability reported in the literature. Intra-rater ICC data are comparable to the upper range of previously reported reliability^12^; however, the present study demonstrated narrower 95% CIs, indicating greater precision and reduced uncertainty around the reliability estimates.

Beyond ICC, measurement tools must detect meaningful change, assessed using SEM and MDC. With radiology often being considered the gold standard, comparisons with imaging provide interesting results. Weight-bearing CT studies report navicular SEM values of 0.66 to 0.80 mm and MDCs around 1.84 to 2.21 mm.^31^ The navicular drop test, a simpler clinical alternative, shows similar SEMs (intra-rater: 0.63-0.781 mm) and MDCs (intra-rater: 1.746-2.165 mm).^28^ Despite a similarity of values, variability occurs between the source of measure including the time intervals used. Often navicular drop test measures variability over short time periods, whereas CT measures repeated static images, not account for image acquisition or repositioning variability.^33^

In contrast, the present US-NH method, especially after CS2 refinements, demonstrated lower SEM and MDC values for intra-rater (0.215 mm, 0.595 mm) and inter-rater (0.403 mm, 1.117 mm), respectively, suggesting changes >~1 mm could represent real change. This precision exceeds navicular drop test and imaging measures, highlighting ultrasonography’s sensitivity to subtle NH changes. Detecting such changes is clinically relevant, as asymmetry may raise lower limb injury risk^34^ and guide rehabilitation targets. However, clinical significance of very small changes remains uncertain and subject to further investigation as literature has reported statistically significant day-to-day differences in plantar navicular displacement of 0.64 ± 1.13 mm, indicating natural fluctuation,^30^ and consequently questions how much change is clinically meaningful vs physiological variability.

Despite improvements, isolated outliers remained in both cohorts, especially in higher arches, possibly because of anatomical variation, deeper soft tissue, or difficulty delineating bone contours using high-frequency ultrasonography. The Clarius L15 probe (5-15 MHz), although suitable for superficial imaging, may have reduced performance in individuals with thicker soft tissue.^35^

### Cohort Influence

The improved ICC, SEM, and MDC likely reflect methodologic refinements and demographic differences. CS1 included a smaller population sample with greater age and BMI—both factors that can increase biological variation in navicular position and surrounding tissues.^36^ Interestingly, closer agreement was observed between US-NH and NH in CS2 (Figure S1C), differing by only 0.36 mm compared with 6 mm, highlighting the potential influence of participant characteristics or tester effects. Although direct comparisons are limited by field of view differences, Shinohara et al^15,16^ (2019, 2022) also reported high correlations (*r* = 0.9, *P* < .001) between sagittal plane ultrasonography and caliper-NH measures.

### Validity

The validity of US-NH was supported by the cadaveric study using direct needle height measurements on the navicular’s surface as a gold standard. Strong correlations were observed between ultrasonography, palpated NH, and direct cadaveric measurements (*r* = 0.98-0.99), indicating consistent relative accuracy. No significant difference was found between US-NH and direct measurements (mean difference = 0.95 mm), demonstrating good agreement between ultrasonographic assessment and anatomical truth. The underestimation of cadaveric US-NH (−3.88 mm, n = 14), compared to participant NH (−2.67 mm), may be attributed to soft tissue compression between fresh frozen and living tissue.^37^ However, the lower US-NH compared with palpated NH aligns with identifying the lower boundary of the navicular rather than the tuberosity, as morphologic studies show landmark differences by shape classification.^10^

### Clinical Implications

This study shows that US-NH is a reliable and precise tool for assessing MLA height, especially when using refinements like gel spacers and transducer stabilisation. In sports and rehabilitation settings, MLA height is important for identifying injury risk and tracking recovery. Although US-NH slightly underestimates navicular height compared to CT/MRI, the difference is minimal in young, healthy populations, making it a practical, radiation-free alternative. The SEM values observed in the present study (0.108-0.466 mm) indicate high measurement precision and are comparable to or lower than those reported for established clinical measures of arch height (~0.56-1.0 mm).^11,38^ The corresponding MDC values (0.3-1.29 mm) therefore may reflect small but real detectable changes in MLA height beyond measurement error. As participants were required to step off and reposition between trials, SEM and MDC values additionally incorporate within-session biological and repositioning variability, providing conservative estimates of repeatability under clinically realistic conditions. Given that reported arch height changes with weightbearing are substantially larger (~5-10 mm),^38^ the present findings suggest the method is sufficiently sensitive to detect changes beyond measurement error, although clinical significance remains to be established in the absence of minimal clinically important difference values for healthy participants.

### Limitations

Several limitations should be considered when interpreting these findings. Although the current protocol demonstrated good inter-rater reliability under controlled conditions, as observed across CS1 and CS2, variability may be greater in routine clinical practice because of differences in operator experience, training standardisation, and adherence to the acquisition protocol. Although the increasing availability and portability of handheld ultrasonography devices may enhance feasibility, appropriate training and standardised procedures will remain essential to maintain measurement reliability in clinical settings. The method requires a controlled acquisition setup, including fixed probe positioning, standardised foot alignment, and post-processing, which may be further streamlined in future through automated analysis approaches but currently limits its use to controlled clinical and research environments. The high-frequency probe, selected for image resolution, may also be limited to individuals with thicker soft tissue or high arches, and soft tissue compression under probe pressure may influence measurements; therefore, future work should consider optimisation of both probe frequency and applied force.

The high-frequency probe, selected for width, may be limited in individuals with thick, soft tissue or high arches. Soft tissue compression under probe pressure may also influence measurements such that future work may wish to focus on both probe-frequency and probe-force. The use of this device in clinical populations, including individuals with foot pathology, obesity, or older adults, should be considered in terms of ease of instruction, probe positioning due to its thickness, and potential challenges in visualising the inferior navicular surface where midfoot fat pad thickness^39^ or arch heights exceed optimal ultrasonographic imaging depth. Despite improved protocols, ultrasonography remains operator-dependent and small shifts in probe angle/tilt during set up, especially over complex contours^40^ can introduce variability. Although portable devices like the Clarius offers portability and accessibility, it lacks fixed anatomical referencing, which can exacerbate variability without careful protocolisation and operator training. Furthermore, no direct comparison with weight-bearing CT or in vivo imaging was undertaken, and although cadaveric validation provides an anatomical reference standard, the use of fresh frozen specimens and a small sample size may limit generalisability because of potential differences in soft tissue properties compared with in vivo conditions.

## Conclusion

This study demonstrates that US-NH can achieve excellent reliability and strong validity when performed using refined protocols, even by operators with limited ultrasonography experience. Methodologic enhancements substantially improved measurement precision, reducing intra- and inter-rater variability and lowering thresholds for detecting real anatomical changes. Although US-NH consistently measured slightly lower palpated NH, its strong correlation with cadaveric measurements supports its potential as an objective tool for assessing MLA morphology. However, successful clinical adoption will depend on standardised protocols and comprehensive training to mitigate operator-dependent variability and assessment with a larger heterogenous cohort.

## Supplemental Material

sj-pdf-1-fao-10.1177_24730114261460463 – Supplemental material for Navicular Height by Weight-bearing Ultrasound: A Reliable and Valid Tool for Assessing the Medial Longitudinal Arch in Physically Active Adults Supplemental material, sj-pdf-1-fao-10.1177_24730114261460463 for Navicular Height by Weight-bearing Ultrasound: A Reliable and Valid Tool for Assessing the Medial Longitudinal Arch in Physically Active Adults by Natasha Noel-Barker, Charles Hillman, Ellys Pollon, Elizabeth Connors, Cameron Christie, William Pettitt, Jack Gallagher, Kathryn Higgins, Molly Riley, Thomas Bestwick-Stevenson and Stefan Kluzek in Foot & Ankle Orthopaedics

## Figures and Tables

**Figure S1A.**
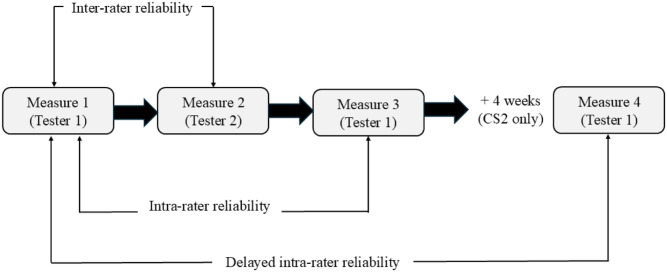
Demonstrating the sequential order of testing for both inter- and intra-rater reliability across 4 individual measures. Abbreviations: CS1, cohort 1; CS2, cohort 2.

**Figure S2A.**
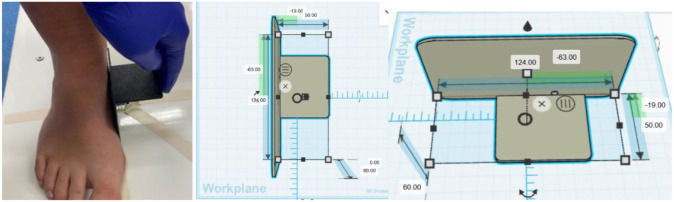
This 3D-printed model was used in cohort 2 (CS2) to help keep the gel within the arch of the standing participant. The integration of this was designed to reduce the number of air bubbles and low acquisition of the image. The left image demonstrates the use with a participant standing on the custom ultrasonography step. The image on the right demonstrates Tinkercad workspace with dimensions. The print file is available on request.

**Figure S2B.**
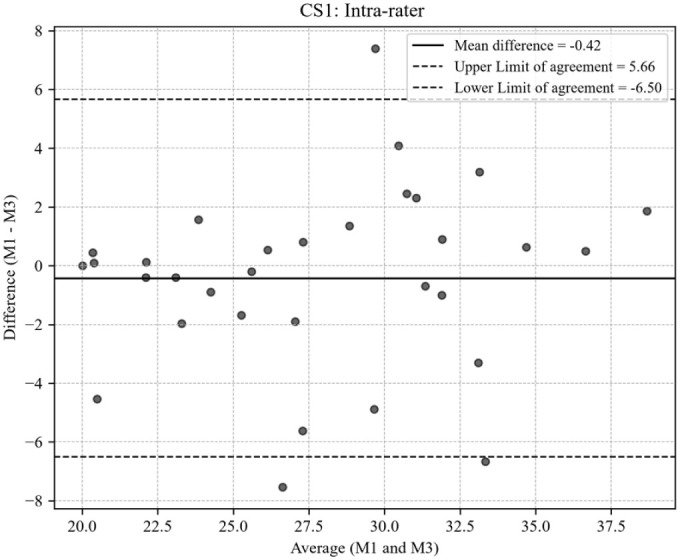
Bland-Altman plot showing intra-rater reliability of US-NH measures (mm) as recorded by Tester 1 (measures 1 and 3) in Cohort 2 (CS2).

**Figure S1B.**
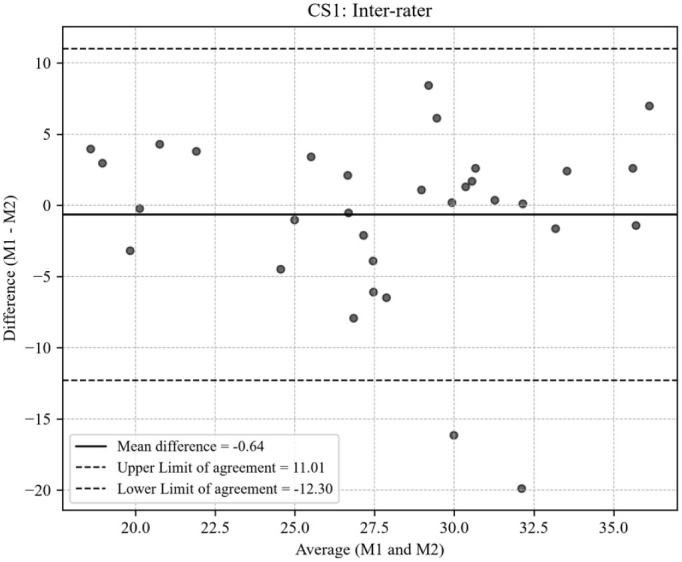
Bland-Altman plot showing intra-rater reliability of US-NH measures (mm) as recorded by Tester 1 (measures 1 and 3), in Cohort 1 (CS1).

**Figure S1C.**
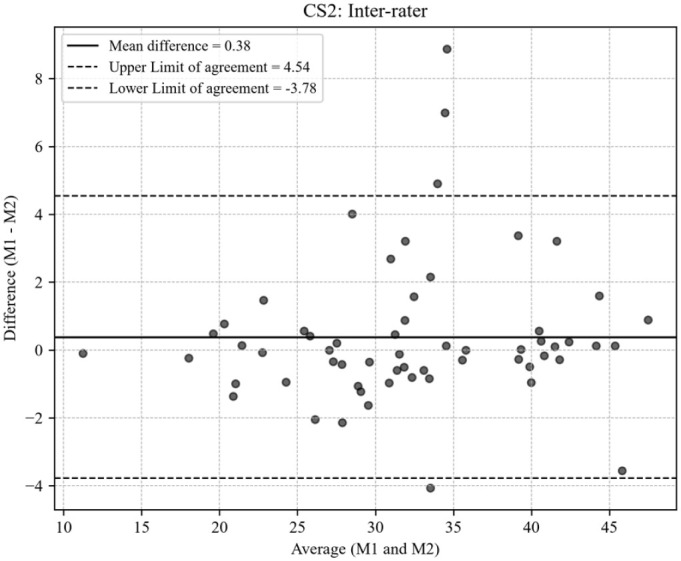
The correlations observed between palpated navicular height (NH) and US-NH (M1 = measurement 1) in Cohort 1 (Left) and Cohort 2 (Right).

## Supplementary Material

### Supplement A

**Table S1A.** Measurements Collected and a Description of the Method.

Measure	Units	Data Collection Method
Foot length	mm	Measured from heel to tip of longest toe using a Brannock device.
Forefoot width	mm	Measured across the widest part of the forefoot using callipers.
Palpated navicular height (NH)	mm	Vertical distance from navicular tuberosity to floor in using metal ruler. Palpated method as described by Menz and Munteanu (2005).^20^
Truncated foot length (TFL)	mm	Distance from heel to first metatarsophalangeal joint using a ruler.
Ratio of navicular height/TFL	ratio	Navicular height/Truncated foot length
Foot Posture Index-6 (FPI-6)	Score (−12 to +12)	Observational scoring of 6-foot posture criteria in standing including. 1. talar head palpation 2. supra and infra lateral malleolar curvature 3. calcaneal inversion or eversion 4. talonavicular joint bulging 5. medial longitudinal arch congruence 6. forefoot abduction or adduction.
Hallux valgus angle	degrees (°)	Measured with a goniometer in semi-weight-bearing whilst the participant was seated with the knee at 100°. The goniometer was aligned to the first MTPJ and the moveable arm to the hallux.
Passive dorsiflexion range	degrees (°)	Measured with a goniometer in semi-weight-bearing whilst the participant was seated with the knee at 100°
Passive first MTPJ dorsiflexion range	degrees (°)	Measured with a goniometer in semi-weight-bearing whilst the participant was seated with the knee at 100°
Active dorsiflexion range	degrees (°)	Measured with a goniometer in semi-weight bearing whilst the participant was seated with the knee at 100°
Active first MTPJ dorsiflexion range	degrees (°)	Measured with a goniometer in semi-weight-bearing whilst the participant was seated with the knee at 100°

Abbreviations: FPI-6, Foot Posture Index-6; MTPJ, metatarsophalangeal Joint; NH, navicular height (palpated); TFL, truncated foot length.

**Table S2A.** Custom-Step Components.

Material/Component	Dimensions/Specifications	URL
RIP-X step platforms	2 steps used with dimensions ‎300 × 800 × 100 mm; 4.2 kg	
Opaque plastic cover	Polypropylene natural plastic sheet 650 × 800 × 10 mm	Fisher Scientific
Ultrasound port	Integrated at standing surface. 70 × 20 mm	
Clarius PAL HD Phased Linear Array probe	4-15 MHz; 50 mm depth; 165 mm width	Clarius
Steel Lab Jack Stand Table (Fisher Scientific)	Not specified (used to mount the probe)	Fisher Scientific
Orbital-Genie non-slip mat (Fisher Scientific)	12.7 mm thick	Fisher Scientific
Lenovo TB-J606F tablet	Android 11	Lenovo
Skintact Ultrasonic Gel (Fanin Ltd, UK)	Used as coupling medium	Fanin Ltd
Silicone moulding filler (PP20-B) *CS2 only*	Applied circumferentially (no specific dimensions given)	Fanin Ltd
Custom 3D-printed gel spacer (“scoop”) *CS2 only*	Please see supplement for detailed measures and 3D printing file	

Abbreviations: CS2, cohort 2; PP, polypropylene; RIP-X, RIP-X step platform.

### Standard Operating Procedure

#### Purpose

To detail the standardized procedure for assembling and utilizing a custom ultrasonography step platform for capturing foot and arch images during participant weight-bearing.

#### Scope

This SOP applies to all personnel involved in the setup and operation of the custom ultrasonography platform in a laboratory or clinical setting.

#### Responsibilities

- Research/Clinical Staff: Responsible for correct assembly, calibration, participant positioning, image acquisition, and hygiene protocol.
- Participants: Follow instructions during the procedure to ensure accurate data collection.

#### Materials and equipment

- As identified in Table A2.

#### Assembly procedure

Platform construction:

○ Stack 3 sections of RIP X steps to the required height.
○ Place an opaque plastic cover over the top, ensuring full coverage.
○ Secure the cover by screwing it into the step frame at multiple points to ensure rigidity.
○ Burr out a port opening from the underside of the step in the desired arch location.

Ultrasound port setup:

○ Ensure the step unit is stable and placed on a secure, flat surface.
○ Secure the probe to the stand using the Genie Orbital Mat, ensuring firm positioning and adjustable alignment.
○ Mount the ultrasound probe underneath the step using the jack lab stand.
○ Use silicone mould putty to cover the gap between the ultrasound transducer surface and the top surface of the platform. This gap is present because of the angle of the probe.

#### Pre-imaging preparation

Sanitization:

○ Clean the opaque surface of the step thoroughly with antibacterial wipes.
○ Ensure no gel residue or dust is present on the step.

Connection Setup:

○ Connect the ultrasound probe to the tablet via Bluetooth.
○ Ensure a clear display is visible for real-time scanning.

Gel Application:

○ Keep ultrasound coupling gel bottle inverted to reduce air bubbles.
○ Apply a small amount of gel directly onto the transducer surface before image capture.

#### Participant setup and imaging procedure

Positioning:

○ Instruct the participant to stand on the platform with the head facing forward, maintaining upright posture.
○ Ask them to align their foot to the long axis line marked on the opaque surface.
○ Using a skin-safe marker, identify and mark the navicular bone.

Alignment:

○ The tester aligns the navicular mark directly above the ultrasound port.
○ Adjust the probe rotation via the lab stand to ensure correct targeting of the ultrasound beam to the navicular.

Imaging:

○ Apply additional gel gently into the arch to improve contact, avoiding the introduction of air bubbles.
○ Begin image capture once bone structures (ie, navicular) are clearly visible on the ultrasonography frame.
○ Ensure the participant remains still during imaging.
○ The 3D-printed gel scoop can be used to keep the ultrasound-medium gel within the participant’s arch. The researcher must place minimal pressure on the gel with only the surface in contact with the participant’s arch.

Between trials:

○ Instruct the participant to step off the platform.
○ Provide them with antibacterial wipes to clean off excess gel.
○ The tester wipes and dries the step platform thoroughly to prevent slippage before subsequent trials.

#### Post-procedure

- Clean all surfaces and equipment thoroughly with antibacterial wipes. Read Table A3.
- Safely store the ultrasound probe and tablet.
- Document session notes, including any anomalies during imaging or participant compliance issues.

#### Protocol amendments

Protocol amendments took place between CS1 and CS2. The main improvement was with image acquisition through use of an adjusting scanning technique.

**Table S3A.** Protocol Amendments Between Cohort 1 and Cohort 2.

Issue observed in CS1	Possible Cause	Action implemented in CS2
Poor acquisition	Air bubbles in gel Gel seeping from arch when participant was standing	Keep transducer gel upside down to remove air bubbles. Slow application into the arch. To maintain gel in the arch, a ‘gel-spacer’ was developed to help contain the gel and maintain full transducer-arch contact. See Figure S2A.
Probe not flush with surface	Jack-lab stand height misaligned	Re-adjust jack-lab stand and mat. Silicone putty reapplied between participants.
Participant slippery surface	Misalignment or excessive gel	‘Gel spacer’ helped guide gel into the participants arch and prevented escape of excess gel. Use of non-slip mat.

Abbreviations: CS1, cohort 1; CS2, cohort 2.

### Custom 3D-Printed Gel Scoop

The 3D-printed gel scoop was developed using Autodesk Tinkercad (https://www.tinkercad.com/). The dimensions can be found in Figure S2A.

### A4: Sanitisation process

Between each participant, the following steps will be performed to ensure hygiene, safety, and accuracy of measurements:

#### Glove change

○ The examiner will discard used gloves and wear a fresh pair of disposable gloves before handling the ultrasound probe or touching the participant.

#### Surface sanitisation

○ The ultrasound probe surface integrated into the measurement device will be thoroughly cleaned using an appropriate disinfectant wipe (eg, 70% isopropyl alcohol or a hospital-grade disinfectant compatible with the probe surface).
○ Allow the surface to air-dry completely to ensure effectiveness and to avoid contamination.

#### Gel application and removal

○ Ultrasound gel will be applied directly to the probe surface and between the participant’s skin and the platform as required.
○ After measurement, any residual gel on the probe surface or the participant’s skin will be removed promptly using a disposable tissue or wipe. This will be disposed of correctly.
○ The step surfaces will be inspected to ensure it is free of gel to prevent slipperiness that might affect measurement accuracy or participant safety when stepping on or off the platform.

#### Step or platform sanitisation

○ Following initial use and between each participant, the step/platform surface will be cleaned using a disinfectant wipe (70% isopropyl alcohol or a hospital-grade disinfectant compatible with the probe surface) and allowed to dry before the next participant.

#### Hand hygiene

○ The examiner will perform hand hygiene by washing hands before donning new gloves and after glove removal.

#### Equipment inspection

○ The probe and device will be regularly inspected for any damage or wear that could compromise hygiene or measurement accuracy.

### Supplement C: Correlations Between Measurement Techniques

Ultrasonography navicular height (US-NH) vs palpated NH measures between cohorts
