# Supplementary material for: Navicular Height by Weight-bearing Ultrasound: A Reliable and Valid Tool for Assessing the Medial Longitudinal Arch in Physically Active Adults
Source: Foot Ankle Orthop. 2026 Jul 28;11(3):24730114261460463. doi: 10.1177/24730114261460463 (PMC13420137; doi:10.1177/24730114261460463)
Supplement: sj-pdf-1-fao-10.1177_24730114261460463 – Supplemental material for Navicular Height by Weight-bearing Ultrasound: A Reliable and Valid Tool for Assessing the Medial Longitudinal Arch in Physically Active Adults [file sj-pdf-1-fao-10.1177_24730114261460463.pdf]

Conflict of Interest (COI) Disclosure Form FAI / FAO

Purpose:
All authors submitting a manuscript to this peer-reviewed journal **must disclose any conflicts of interest (COI) related to the submitted paper**.
No other disclosures are required or desired.

A conflict of interest typically involves **financial relationships (most commonly payments to surgeons or authors)** that could reasonably be perceived as influencing the submitted work, whether **received in the past or anticipated in the future**.

**Author Name:**	**Natasha Noel-Barker**
**Manuscript Title:**	**Navicular Height by Weight-bearing Ultrasound: A Reliable and Valid Tool for Assessing the Medial Longitudinal Arch in Physically Active Adults**
**Date:**	**02/02/2026**

Conflict of Interest Disclosure:

Do you or a family member have conflicts of interest related to this manuscript or its subject matter?
(Examples include consulting fees, honoraria, royalties, stock or stock options, research funding, paid advisory roles, product or material support, or intellectual property interests (e.g., potential future royalties) valued over $500, received within the past three years or expected in the future.)

☒ **No — I have no conflicts of interest to disclose.**

☐ **Yes — I have a conflict of interest to disclose.**
If **Yes**, please briefly describe the financial relationship(s) below, including the company/entity involved and the nature of the payment or support:

Click or tap here to enter text.

Updates: If a relevant conflict of interest arises or changes after submission and before publication, an updated disclosure must be provided.

Publication of Disclosure: All disclosed conflicts of interest will be published with the article and completed forms will be made available as an online supplement.

Conflict of Interest (COI) Disclosure Form FAI / FAO

Purpose:
All authors submitting a manuscript to this peer-reviewed journal **must disclose any conflicts of interest (COI) related to the submitted paper**.
No other disclosures are required or desired.

A conflict of interest typically involves **financial relationships (most commonly payments to surgeons or authors)** that could reasonably be perceived as influencing the submitted work, whether **received in the past or anticipated in the future**.

**Author Name:**	**Charles Hillman**
**Manuscript Title:**	**Navicular Height by Weight-bearing Ultrasound: A Reliable and Valid Tool for Assessing the Medial Longitudinal Arch in Physically Active Adults**
**Date:**	**02/02/2026**

Conflict of Interest Disclosure:

Do you or a family member have conflicts of interest related to this manuscript or its subject matter?
(Examples include consulting fees, honoraria, royalties, stock or stock options, research funding, paid advisory roles, product or material support, or intellectual property interests (e.g., potential future royalties) valued over $500, received within the past three years or expected in the future.)

☒ **No — I have no conflicts of interest to disclose.**

☐ **Yes — I have a conflict of interest to disclose.**
If **Yes**, please briefly describe the financial relationship(s) below, including the company/entity involved and the nature of the payment or support:

Click or tap here to enter text.

Updates: If a relevant conflict of interest arises or changes after submission and before publication, an updated disclosure must be provided.

Publication of Disclosure: All disclosed conflicts of interest will be published with the article and completed forms will be made available as an online supplement.

Conflict of Interest (COI) Disclosure Form FAI / FAO

Purpose:
All authors submitting a manuscript to this peer-reviewed journal **must disclose any conflicts of interest (COI) related to the submitted paper**.
No other disclosures are required or desired.

A conflict of interest typically involves **financial relationships (most commonly payments to surgeons or authors)** that could reasonably be perceived as influencing the submitted work, whether **received in the past or anticipated in the future**.

**Author Name:**	**Ellys Pollon**
**Manuscript Title:**	**Navicular Height by Weight-bearing Ultrasound: A Reliable and Valid Tool for Assessing the Medial Longitudinal Arch in Physically Active Adults**
**Date:**	**02/02/2026**

Conflict of Interest Disclosure:

Do you or a family member have conflicts of interest related to this manuscript or its subject matter?
(Examples include consulting fees, honoraria, royalties, stock or stock options, research funding, paid advisory roles, product or material support, or intellectual property interests (e.g., potential future royalties) valued over $500, received within the past three years or expected in the future.)

☒ **No — I have no conflicts of interest to disclose.**

☐ **Yes — I have a conflict of interest to disclose.**
If **Yes**, please briefly describe the financial relationship(s) below, including the company/entity involved and the nature of the payment or support:

Click or tap here to enter text.

Updates: If a relevant conflict of interest arises or changes after submission and before publication, an updated disclosure must be provided.

Publication of Disclosure: All disclosed conflicts of interest will be published with the article and completed forms will be made available as an online supplement.

Conflict of Interest (COI) Disclosure Form FAI / FAO

Purpose:
All authors submitting a manuscript to this peer-reviewed journal **must disclose any conflicts of interest (COI) related to the submitted paper**.
No other disclosures are required or desired.

A conflict of interest typically involves **financial relationships (most commonly payments to surgeons or authors)** that could reasonably be perceived as influencing the submitted work, whether **received in the past or anticipated in the future**.

**Author Name:**	**Elizabeth Connors**
**Manuscript Title:**	**Navicular Height by Weight-bearing Ultrasound: A Reliable and Valid Tool for Assessing the Medial Longitudinal Arch in Physically Active Adults**
**Date:**	**02/02/2026**

Conflict of Interest Disclosure:

Do you or a family member have conflicts of interest related to this manuscript or its subject matter?
(Examples include consulting fees, honoraria, royalties, stock or stock options, research funding, paid advisory roles, product or material support, or intellectual property interests (e.g., potential future royalties) valued over $500, received within the past three years or expected in the future.)

☒ **No — I have no conflicts of interest to disclose.**

☐ **Yes — I have a conflict of interest to disclose.**
If **Yes**, please briefly describe the financial relationship(s) below, including the company/entity involved and the nature of the payment or support:

Click or tap here to enter text.

Updates: If a relevant conflict of interest arises or changes after submission and before publication, an updated disclosure must be provided.

Publication of Disclosure: All disclosed conflicts of interest will be published with the article and completed forms will be made available as an online supplement.

Conflict of Interest (COI) Disclosure Form FAI / FAO

Purpose:
All authors submitting a manuscript to this peer-reviewed journal **must disclose any conflicts of interest (COI) related to the submitted paper**.
No other disclosures are required or desired.

A conflict of interest typically involves **financial relationships (most commonly payments to surgeons or authors)** that could reasonably be perceived as influencing the submitted work, whether **received in the past or anticipated in the future**.

**Author Name:**	**Cameron Christie**
**Manuscript Title:**	**Navicular Height by Weight-bearing Ultrasound: A Reliable and Valid Tool for Assessing the Medial Longitudinal Arch in Physically Active Adults**
**Date:**	**02/02/2026**

Conflict of Interest Disclosure:

Do you or a family member have conflicts of interest related to this manuscript or its subject matter?
(Examples include consulting fees, honoraria, royalties, stock or stock options, research funding, paid advisory roles, product or material support, or intellectual property interests (e.g., potential future royalties) valued over $500, received within the past three years or expected in the future.)

☒ **No — I have no conflicts of interest to disclose.**

☐ **Yes — I have a conflict of interest to disclose.**
If **Yes**, please briefly describe the financial relationship(s) below, including the company/entity involved and the nature of the payment or support:

Click or tap here to enter text.

Updates: If a relevant conflict of interest arises or changes after submission and before publication, an updated disclosure must be provided.

Publication of Disclosure: All disclosed conflicts of interest will be published with the article and completed forms will be made available as an online supplement.

Conflict of Interest (COI) Disclosure Form FAI / FAO

Purpose:
All authors submitting a manuscript to this peer-reviewed journal **must disclose any conflicts of interest (COI) related to the submitted paper**.
No other disclosures are required or desired.

A conflict of interest typically involves **financial relationships (most commonly payments to surgeons or authors)** that could reasonably be perceived as influencing the submitted work, whether **received in the past or anticipated in the future**.

**Author Name:**	**William Petit**
**Manuscript Title:**	**Navicular Height by Weight-bearing Ultrasound: A Reliable and Valid Tool for Assessing the Medial Longitudinal Arch in Physically Active Adults**
**Date:**	**02/02/2026**

Conflict of Interest Disclosure:

Do you or a family member have conflicts of interest related to this manuscript or its subject matter?
(Examples include consulting fees, honoraria, royalties, stock or stock options, research funding, paid advisory roles, product or material support, or intellectual property interests (e.g., potential future royalties) valued over $500, received within the past three years or expected in the future.)

☒ **No — I have no conflicts of interest to disclose.**

☐ **Yes — I have a conflict of interest to disclose.**
If **Yes**, please briefly describe the financial relationship(s) below, including the company/entity involved and the nature of the payment or support:

Click or tap here to enter text.

Updates: If a relevant conflict of interest arises or changes after submission and before publication, an updated disclosure must be provided.

Publication of Disclosure: All disclosed conflicts of interest will be published with the article and completed forms will be made available as an online supplement.

Conflict of Interest (COI) Disclosure Form FAI / FAO

Purpose:
All authors submitting a manuscript to this peer-reviewed journal **must disclose any conflicts of interest (COI) related to the submitted paper**.
No other disclosures are required or desired.

A conflict of interest typically involves **financial relationships (most commonly payments to surgeons or authors)** that could reasonably be perceived as influencing the submitted work, whether **received in the past or anticipated in the future**.

**Author Name:**	**Jack Gallagher**
**Manuscript Title:**	**Navicular Height by Weight-bearing Ultrasound: A Reliable and Valid Tool for Assessing the Medial Longitudinal Arch in Physically Active Adults**
**Date:**	**02/02/2026**

Conflict of Interest Disclosure:

Do you or a family member have conflicts of interest related to this manuscript or its subject matter?
(Examples include consulting fees, honoraria, royalties, stock or stock options, research funding, paid advisory roles, product or material support, or intellectual property interests (e.g., potential future royalties) valued over $500, received within the past three years or expected in the future.)

☒ **No — I have no conflicts of interest to disclose.**

☐ **Yes — I have a conflict of interest to disclose.**
If **Yes**, please briefly describe the financial relationship(s) below, including the company/entity involved and the nature of the payment or support:

Click or tap here to enter text.

Updates: If a relevant conflict of interest arises or changes after submission and before publication, an updated disclosure must be provided.

Publication of Disclosure: All disclosed conflicts of interest will be published with the article and completed forms will be made available as an online supplement.

Conflict of Interest (COI) Disclosure Form FAI / FAO

Purpose:
All authors submitting a manuscript to this peer-reviewed journal **must disclose any conflicts of interest (COI) related to the submitted paper**.
No other disclosures are required or desired.

A conflict of interest typically involves **financial relationships (most commonly payments to surgeons or authors)** that could reasonably be perceived as influencing the submitted work, whether **received in the past or anticipated in the future**.

**Author Name:**	**Kathryn Higgins**
**Manuscript Title:**	**Navicular Height by Weight-bearing Ultrasound: A Reliable and Valid Tool for Assessing the Medial Longitudinal Arch in Physically Active Adults**
**Date:**	**02/02/2026**

Conflict of Interest Disclosure:

Do you or a family member have conflicts of interest related to this manuscript or its subject matter?
(Examples include consulting fees, honoraria, royalties, stock or stock options, research funding, paid advisory roles, product or material support, or intellectual property interests (e.g., potential future royalties) valued over $500, received within the past three years or expected in the future.)

☒ **No — I have no conflicts of interest to disclose.**

☐ **Yes — I have a conflict of interest to disclose.**
If **Yes**, please briefly describe the financial relationship(s) below, including the company/entity involved and the nature of the payment or support:

Click or tap here to enter text.

Updates: If a relevant conflict of interest arises or changes after submission and before publication, an updated disclosure must be provided.

Publication of Disclosure: All disclosed conflicts of interest will be published with the article and completed forms will be made available as an online supplement.

Conflict of Interest (COI) Disclosure Form FAI / FAO

Purpose:
All authors submitting a manuscript to this peer-reviewed journal **must disclose any conflicts of interest (COI) related to the submitted paper**.
No other disclosures are required or desired.

A conflict of interest typically involves **financial relationships (most commonly payments to surgeons or authors)** that could reasonably be perceived as influencing the submitted work, whether **received in the past or anticipated in the future**.

**Author Name:**	**Molly Riley**
**Manuscript Title:**	**Navicular Height by Weight-bearing Ultrasound: A Reliable and Valid Tool for Assessing the Medial Longitudinal Arch in Physically Active Adults**
**Date:**	**02/02/2026**

Conflict of Interest Disclosure:

Do you or a family member have conflicts of interest related to this manuscript or its subject matter?
(Examples include consulting fees, honoraria, royalties, stock or stock options, research funding, paid advisory roles, product or material support, or intellectual property interests (e.g., potential future royalties) valued over $500, received within the past three years or expected in the future.)

☒ **No — I have no conflicts of interest to disclose.**

☐ **Yes — I have a conflict of interest to disclose.**
If **Yes**, please briefly describe the financial relationship(s) below, including the company/entity involved and the nature of the payment or support:

Click or tap here to enter text.

Updates: If a relevant conflict of interest arises or changes after submission and before publication, an updated disclosure must be provided.

Publication of Disclosure: All disclosed conflicts of interest will be published with the article and completed forms will be made available as an online supplement.

Conflict of Interest (COI) Disclosure Form FAI / FAO

Purpose:
All authors submitting a manuscript to this peer-reviewed journal **must disclose any conflicts of interest (COI) related to the submitted paper**.
No other disclosures are required or desired.

A conflict of interest typically involves **financial relationships (most commonly payments to surgeons or authors)** that could reasonably be perceived as influencing the submitted work, whether **received in the past or anticipated in the future**.

**Author Name:**	**Thomas Bestwick-Stevenson**
**Manuscript Title:**	**Navicular Height by Weight-bearing Ultrasound: A Reliable and Valid Tool for Assessing the Medial Longitudinal Arch in Physically Active Adults**
**Date:**	**02/02/2026**

Conflict of Interest Disclosure:

Do you or a family member have conflicts of interest related to this manuscript or its subject matter?
(Examples include consulting fees, honoraria, royalties, stock or stock options, research funding, paid advisory roles, product or material support, or intellectual property interests (e.g., potential future royalties) valued over $500, received within the past three years or expected in the future.)

☒ **No — I have no conflicts of interest to disclose.**

☐ **Yes — I have a conflict of interest to disclose.**
If **Yes**, please briefly describe the financial relationship(s) below, including the company/entity involved and the nature of the payment or support:

Click or tap here to enter text.

Updates: If a relevant conflict of interest arises or changes after submission and before publication, an updated disclosure must be provided.

Publication of Disclosure: All disclosed conflicts of interest will be published with the article and completed forms will be made available as an online supplement.

Conflict of Interest (COI) Disclosure Form FAI / FAO

Purpose:
All authors submitting a manuscript to this peer-reviewed journal **must disclose any conflicts of interest (COI) related to the submitted paper**.
No other disclosures are required or desired.

A conflict of interest typically involves **financial relationships (most commonly payments to surgeons or authors)** that could reasonably be perceived as influencing the submitted work, whether **received in the past or anticipated in the future**.

**Author Name:**	**Stefan Kluzek**
**Manuscript Title:**	**Navicular Height by Weight-bearing Ultrasound: A Reliable and Valid Tool for Assessing the Medial Longitudinal Arch in Physically Active Adults**
**Date:**	**02/02/2026**

Conflict of Interest Disclosure:

Do you or a family member have conflicts of interest related to this manuscript or its subject matter?
(Examples include consulting fees, honoraria, royalties, stock or stock options, research funding, paid advisory roles, product or material support, or intellectual property interests (e.g., potential future royalties) valued over $500, received within the past three years or expected in the future.)

☒ **No — I have no conflicts of interest to disclose.**

☐ **Yes — I have a conflict of interest to disclose.**
If **Yes**, please briefly describe the financial relationship(s) below, including the company/entity involved and the nature of the payment or support:

Click or tap here to enter text.

Updates: If a relevant conflict of interest arises or changes after submission and before publication, an updated disclosure must be provided.

Publication of Disclosure: All disclosed conflicts of interest will be published with the article and completed forms will be made available as an online supplement.
